# Developing an Analytical Framework for the Molecular Weight Analysis of Fish‐Derived Collagen Peptides in Beverages: A Multi‐Method Comparative Study

**DOI:** 10.1002/fsn3.72386

**Published:** 2026-09-18

**Authors:** Mengmeng Feng, Yali Li, Yongkang Luo, Yanfei Jiang, Chunyue Zhao

**Affiliations:** ^1^ Beijing Qingyan Boshi Health Management Co., Ltd Beijing China; ^2^ China Agricultural University Beijing China

**Keywords:** curve fitting, fish‐derived collagen peptide, functional beverage, gel permeation chromatography, molecular weight parameter

## Abstract

Accurate determination of the molecular weight of collagen peptides is crucial for quality control in functional beverages. Current national and industry standard methods for peptide powders face challenges of matrix interference and insufficient comparability when applied to complex beverage systems. In this study, three mainstream chromatographic methods were systematically evaluated for their suitability, reproducibility, and consistency in determining the molecular weight (MW) of fish‐derived collagen peptides in beverages. An analytical framework was established by identifying and removing interference peaks from non‐peptide excipients, followed by systematic comparison of linear and cubic fitting models. All three methods exhibited good reproducibility. After interference peak removal, the peptide profiles of the beverages were highly consistent with those of pure peptide powder. Cubic fitting reduced inter‐method differences, with relative standard deviation (RSD) values below 5% in the bioactive < 1 kDa range. The framework was further validated through beverage formulation optimization, demonstrating compatibility between functional ingredient quality control and sensory flavor adjustability. The proposed approach provides a practical solution for industry quality control and cross‐laboratory data comparability of collagen peptide beverages.

## Graphical abstract

An analytical framework was developed for molecular weight analysis of fish‐derived collagen peptides in beverages. Three GPC methods were compared, and matrix interference from excipients was excluded using a 20.5 min cutoff. Cubic polynomial fitting significantly improved inter‐method consistency. The framework was validated during beverage formulation optimization, ensuring peptide quality control while maintaining sensory flavor. This provides a practical strategy for industrial quality control and cross‐laboratory data comparability.

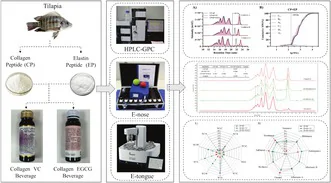

## 1 Introduction

With rising living standards and an aging population, counteracting skin aging has become a widely recognized health concern (He et al. 2023). The main manifestations of skin aging include dryness, sagging, pigmentation, telangiectasia, and wrinkles caused by the loss of collagen and elastic fibers, with mechanisms involving free radicals and oxidative stress, inflammatory responses, and non‐enzymatic glycosylation (Wong and Chew 2021). In the beauty and health industries, the market demand for anti‐aging products continues to grow, among which oral or topical supplementation of collagen and its related active ingredients has become an important approach (Dorf and Maciejczyk 2024; Furman et al. 2025; Patel et al. 2024). Fish‐derived small peptides, mainly produced through enzymatic hydrolysis of by‐products such as fish skin, scales, and bones, are widely used in functional foods, health products, and cosmetics due to their low allergenicity, high bioavailability, and diverse bioactivities, including antioxidant, anti‐inflammatory, anti‐aging, and tissue‐repair effects (Baraiya et al. 2024; Luo et al. 2020; Zhao et al. 2021).

Collagen and elastin are core structural proteins that maintain skin integrity and functionality. Together, they form the extracellular matrix network, providing mechanical strength and elastic resilience (Bielajew et al. 2020; Diller and Tabor 2022; Liu et al. 2019). Exogenous collagen peptides can regulate the skin microenvironment, improving skin condition and delaying photoaging by promoting fibroblast activity and increasing hyaluronic acid synthesis (Cho et al. 2023). Moreover, collagen peptides protect the extracellular matrix from degradation by inhibiting the release of matrix metalloproteinases MMP‐1 and MMP‐3, thereby maintaining structural stability (Edgar et al. 2018). Elastin and its derived peptides stimulate Type I procollagen synthesis, enhance skin elasticity, and exert photoprotective effects by regulating elastase activity (Bai et al. 2024). Nutritional supplements in the anti‐aging field often achieve synergistic effects by combining collagen peptides with vitamins and antioxidants (Izadi et al. 2024; Kaufman et al. 2024). The combined use of collagen and elastin peptides has drawn particular attention due to their complementary roles in enhancing structural integrity and inhibiting matrix degradation (Ali et al. 2025).

Despite the clear potential of collagen and elastin peptides in anti‐aging applications, their bioactivity and product quality heavily depend on the molecular weight distribution, composition, and structure of the peptides (Sánchez and Vázquez 2017; Wu et al. 2025). Our previous study on the collagen peptide beverage confirmed that the molecular weight distribution remained stable during 3 months of accelerated storage, with the fraction below 1 kDa accounting for over 65%, while ABTS radical scavenging activity was maintained at 80%–90% under these conditions (Shi et al. 2025). At present, several national standards and industry methods have been established for the molecular weight determination of fish‐derived collagen peptides, but these methods mainly target single‐ingredient peptide powder raw materials (Feng et al. 2025). In practical applications, peptides are often consumed in complex forms such as beverages, where added vitamins, organic acids, flavoring agents, and other excipients may cause matrix interference in the analytical process. More critically, differences in chromatographic conditions and data‐processing models among various detection methods have not been systematically evaluated for result consistency and comparability, posing challenges for industry quality control and standardization.

Therefore, this study focused on the combined system of collagen and elastin peptides, evaluating the applicability, reproducibility, and result consistency of three mainstream gel permeation chromatography (GPC) methods in beverage matrices. By comparing the effects of different chromatographic conditions and data‐processing models (linear and cubic fitting) on the molecular weight distribution and key parameters, we assessed matrix interference resistance, data comparability, and practical feasibility. The research aims to establish a detection and evaluation procedure suitable for complex beverage systems that supports cross‐platform data comparison and provides methodological foundations for quality control, standardization, and product innovation in collagen peptide functional beverages.

## 2 Materials and Methods

### 2.1 Materials and Reagents

Trifluoroacetic acid (TFA), LC‐grade acetonitrile (ACN), Vitamin C (VC), and epigallocatechin gallate (EGCG) were obtained from Shanghai Aladdin Reagent Co. Ltd., while the standards were purchased from Beijing Solarbio Technology Co. Ltd., including Cytochrome C (12384 Da), Aprotinin (6511.44 Da), Bacitracin (1422.69 Da), L‐oxidized glutathione (L‐GSH, 612.63 Da), Gly‐Gly‐Tyr‐Arg (GGTA, 451.48 Da), Gly‐Pro‐Hyp (GPH, 285.29 Da), and Gly‐Gly‐Gly (GGG, 189 Da). Two commercially available beverages (50 mL/bottle) from Beijing Qingyan Boshi Health Management Co. Ltd. (brand: “Five Doctors”) were used as test matrices and designated as collagen‐VC beverage and collagen‐EGCG beverage, respectively. The collagen‐VC beverage contained fish‐derived collagen peptide (CP) at 5000 mg/50 mL, elastin peptide (EP) at 100 mg/50 mL, and vitamin C (VC) at 100 mg/50 mL. The collagen‐EGCG beverage contained CP at 6000 mg/50 mL, EP at 100 mg/50 mL, EGCG at 100 mg/50 mL, along with sodium hyaluronate, chitosan oligosaccharide, and broccoli seed extract. The CP and EP raw materials used for preparation of blank control samples were provided by Hainan Huayan Collagen Co. Ltd., and were from the same production batches as those in the commercial beverages, ensuring identical peptide sources between blank controls and beverage samples. Blank control samples consisted of the peptide powders only, without excipients.

### 2.2 Molecular Weight Distribution Analysis

#### 2.2.1 Chromatographic Condition

GPC was performed using a HPLC system (LC‐20AT, Shimadzu, Japan) equipped with a diode array detector (SPD‐M20A, Shimadzu), and a TSK‐GEL G2000 SWXL (300 × 7.8 mm, 5 μm; TOSOH, Japan). Three mobile phase compositions, corresponding to different Chinese standards for peptide molecular weight determination, were compared (Feng et al. 2025): acetonitrile/water/TFA = 40/60/0.1 (v/v/v) per GB 31645‐2018; 45/55/0.1 (v/v/v) per GB/T 22729‐2008; and 30/70/0.1 (v/v/v) per Q/HNHY 002‐2021. The flow rate was 0.5 mL/min, the column temperature was maintained at 30°C, and the UV absorbance was monitored at 220 nm. Unlike traditional HPLC, which quantifies based on concentration and peak area, GPC is a semi‐quantitative technique that normalizes the area based on retention time (Held and Kilz 2009). The recommended loading concentrations in these standards differ: 5 mg/mL for GB 31645‐2018 and Q/HNHY 002‐2021, and 2 mg/mL for GB/T 22729‐2008. Given that minor concentration variations do not substantially affect area‐normalized molecular weight distribution as long as the column is not overloaded and the signal remains above the detection limit, a uniform loading concentration of 5 mg/mL was adopted across all three methods in this study. Table 1 summarizes the detailed information of the three standards.

**TABLE 1:** Comparison of chromatographic conditions, calibration parameters, and fitting equations for the three standard methods.

Reference standards	Q/HNHY 002‐2021	GB 31645‐2018	GB/T 22729‐2008
Standard designation	Collagen tripeptide	Collagen peptide	Oligopeptides powder of marine fish
Standard category	Enterprise standard	National standard	Recommended national standard
Referred to as	Method A	Method B	Method C
Calibration standards	Cytochrome C, aprotinin, bacitracin, L‐GSH, GPH, GGG	Cytochrome C, aprotinin, bacitracin, GGTA, GGG	
Mobile phase	ACN/H_2_O/TFA = 30/70/0.1	ACN/H_2_O/TFA = 40:60/0.05	ACN/H_2_O/TFA = 45/55/0.1
Chromatographic column	Japan Tocho TSK‐GEL G2000 SWXL (300 mm × 7.8 mm, 5 μm)		
Other parameters	UV: 220 nm; Flow: 0.5 mL/min; Injection volume: 10 μL; Run time: 30 min		
Loading content	5 mg/mL	5 mg/mL	2 mg/mL
Linear fitting (*R* ^2^, RMSE)	*y* = −0.2465lg(x) + 7.0746 *R* ^2^ = 0.9925, RMSE = 0.0579	*y* = −0.2485lg(x) + 7.0528 *R* ^2^ = 0.9898, RMSE = 0.0689	*y* = −0.2498lg(x) + 7.0327 *R* ^2^ = 0.9798, RMSE = 0.097
Cubic fitting (*R* ^2^, RMSE)	*y* = 0.0043lg(x)^3^–0.2030lg(x)^2^ + 2.9195lg(x)–9.0937 *R* ^2^ = 0.997, RMSE = 0.0367	*y* = 0.0050lg(x)^3^–0.2304lg(x)^2^ + 3.2233lg(x)–10.0731 *R* ^2^ = 0.9999, RMSE = 0.0023	*y* = 0.0060lg(x)^3^–0.2729lg(x)^2^ + 3.7855lg(x)‐12.4738 *R* ^2^ = 0.9982, RMSE = 0.0287

*Note:* ACN, acetonitrile; GGT, Gly‐Gly‐Tyr; GPH, Gly‐Pro‐Hyp; GSH, glutathione (reduced); RMSE, root mean square error; TFA, trifluoroacetic acid.

#### 2.2.2 Sample Preparation

All samples (commercial beverages and the blank controls samples) were processed using the same procedure (Shi et al. 2025). Based on the label‐declared peptide contents, the samples were diluted with each of the three mobile phases described in Section 2.2.1 to a final peptide concentration of 5 mg/mL, ultrasonicated for 10 min, filtered through 0.22 μm membranes (Jinteng, Tianjin), and transferred to HPLC vials. The injection volume was 10 μL. GPC separates analytes by size: larger molecules elute first because they are excluded from the pores of the stationary phase, while smaller molecules penetrate the pores and are retained longer. Molecular weight distributions and their parameters, including number‐average MW (Mn), weight‐average MW (Mw), and viscosity‐average MW (Mv), were calculated by LabSolutions (Version 5.124, Shimadzu, Japan) with a calibration curve constructed from corresponding peptide standards by plotting lg(MW) against retention time, fitted with both linear and cubic fitting. All samples were analyzed in triplicate.

### 2.3 Similarity Evaluation

Chromatographic fingerprint similarity evaluation was performed using the Similarity Evaluation System for Chromatographic Fingerprint of Traditional Chinese Medicines (Version 2012.130723) recommended by the Chinese Pharmacopeia Commission (Cai et al. 2025). The chromatogram of collagen peptide powder was used as the reference fingerprint. The time window width was set to 0.1 min, and peak matching was conducted using the Mark peak matching method following multi‐point calibration, generating a reference fingerprint. Similarities between each sample chromatogram and the reference fingerprint were calculated using the median method.

### 2.4 Flavor Analysis

#### 2.4.1 E‐Nose

Ten metal oxide gas sensor arrays make up the electronic nose (PEN3, AIRSENSE, Germany), which measures aroma values of different substances. The analytical procedure was modified slightly from Chen et al. (2023). A 20 mL glass vial containing 30 mg/mL of collagen powder and beverage was filled and allowed to balance for 30 min at room temperature. An electronic nose probe was fitted to absorb the top gas and identify the aroma compound. Five parallel measurements were analyzed, measuring in parallel for 5 times.

E‐tongue: The electronic tongue (INSENT, SA402B, Japan) has six sensors, which can output 9 indicators, including six basic tastes (Umami, Saltiness, Sourness, Sweetness, Bitterness, Astringency) and three aftertastes (Umami richness, Bitterness aftertaste, Astringency aftertaste). After being pre‐treated for 24 h in a standard solution (30 mM KCl solution with 0.3 mM tartaric acid), the sensor was self‐tested and calibrated (Liang et al. 2022). A 50 mL glass vial containing collagen powder and beverage of 15 mg/mL was vortexed for 10 min, and dissolved and filtered with a 0.45 μm membrane. The last three repetitions of the four replicates for the five basic tastes were valid data, and the sweetness was measured separately in parallel for five repetitions.

### 2.5 Statistical Analysis

All data were expressed as mean ± SD from three independent replicate measurements. Curve fitting (linear and cubic polynomial regression), calculation of coefficients of determination (*R* ^2^) and root mean square error (RMSE), as well as residual analysis, were performed using GraphPad Prism 8 (GraphPad Software, San Diego, CA, USA). All figures were generated using GraphPad Prism 8, Origin 2024 (OriginLab, Northampton, MA, USA), and Microsoft PowerPoint 2016. For inter‐method comparisons of molecular weight distribution and parameters, significant differences were determined based on relative deviation percentage (RD, %). This approach references the requirement commonly found in national standards that the absolute difference between two independent determination results under repeatability conditions shall not exceed 10% of the arithmetic mean. Although this 10% threshold was originally intended for evaluating intra‐method precision, it has been adapted for inter‐method comparisons in this study given its general acceptance in industrial quality control.

## 3 Results

### 3.1 Comparsion of Analytical Methods

#### 3.1.1 Comparison of Calibration Curve Fitting Effects

To systematically evaluate the reliability of the molecular weight characterization methods, three mainstream methods recommended by the Chinese national standards and industry standards were selected (Feng et al. 2025). The main difference lies in the composition of the mobile phase. The standard curves of the three methods are shown in Table 1. The *R* ^2^ of the linear fitting ranges from 0.9925 to 0.9978. After re‐fitting using a cubic polynomial, the *R* ^2^ of the calibration models of the three methods increased to 0.9970 to 0.9999, and the RMSE decreased from 0.0579 to 0.0970 to 0.0023 to 0.0367, with a significant improvement in fitting accuracy. Method B performed the best, with an *R* ^2^ of 0.9999 and an RMSE of only 0.0023.

**FIGURE 1:**
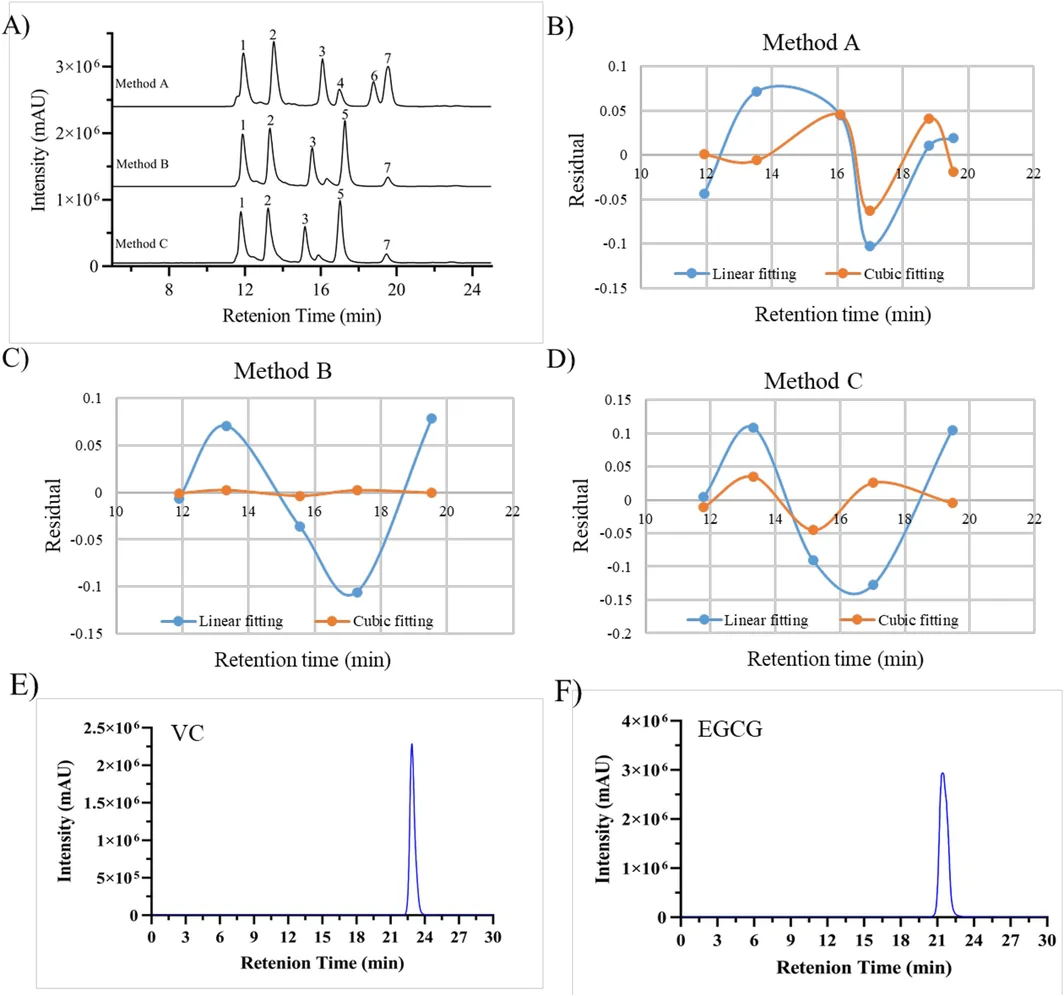
(A) Chromatograms of peptide standards for Methods A, B and C. (B–D) Residual distributions of peptide standards for Methods A, B and C under linear and cubic fitting. (E, F) Chromatograms of VC and EGCG for Method C. The horizontal dashed line at *y* = 0 indicates perfect prediction. Cytochrome C (1), Aprotinin (2), Bacitracin (3), GGTA (4), L‐GSH (5), GPH (6), GGG (7).

Figure 1A shows the chromatograms of the peptide standard samples for the three methods, while Figure 1B–D represent the corresponding standard deviation distributions. The residuals of the linear fitting all show a clear U‐shaped or inverted U‐shaped trend, indicating that there is a systematic deviation in the linear model, and it fails to accurately describe the nonlinear relationship between lg(MW) and retention time. The residuals of the cubic fitting are basically randomly distributed around the zero line, with most residual values being within ±0.05, and the model is relatively reasonable and acceptable; among them, method B has the highest accuracy, with residuals within ±0.01, and method C comes second.

Held and Kilz (2009) also pointed out that the GPC calibration curve is usually S‐shaped rather than linear, so the most commonly used fitting function is a polynomial fitting function. *R* ^2^ is not the only criterion for selecting the optimal fitting function, because even when *R* ^2^ is close to 1, the average deviation can still be quite large. Although higher‐order polynomials can reduce the fitting error, they will lead to meaningless maxima and minima in the first‐order derivative. If the regression coefficient is the only criterion for selecting the fitting function, the GPC result should reach 0.999 and have the smallest error. Considering the *R* ^2^ and RMSE data as well as the residual distribution, the cubic polynomial is significantly superior to linear fitting in the calibration curve fitting stage. Further verification of its applicability will be conducted through the comparison of molecular weight distribution and parameters in the subsequent stage.

#### 3.1.2 Repeatability and Precision Verification

To ensure instrument stability, standard samples were re‐injected before and after each batch of sample analysis. Across all three methods, the RSD of the retention time for standard samples was below 0.1%, indicating that the instrument system was stable and the chromatographic column was in a balanced state (Table S1). At the same time, the theoretical plate number (*N*) of the minimum standard sample GGG (189 Da) was calculated (*N* = 5.54 × (tR/W_1_/_2_)^2^), and the *N* values for the three methods were 11,436, 5924 and 2764 respectively. All of these values were higher than the *N* > 2000 required by the system suitability, proving that the chromatographic column did not age and affect the separation performance. Linear fitting and cubic polynomial fitting methods were used to analyze the molecular weight distribution and related parameters of the peptide powder (composed of collagen and elastin) as well as the two functional beverages (Collagen VC and EGCG beverages) that contained the same amount of small peptide powder. The RSD values of five molecular weight ranges (> 3 kDa, 2–3 kDa, 1–2 kDa, 0.5–1 kDa, < 0.5 kDa) and three key molecular weight parameters (Mn, Mw, Mz) were calculated. The results (Table 2) showed that the RSD of all samples was below 3%, confirming that these three methods have good repeatability and precision, and are suitable for subsequent comparative analysis.

**TABLE 2:** Relative standard deviation (RSD, %) of different molecular weight components in collagen powder and beverages under different mobile phase and fitting condition.

RSD%	Method A			Method B			Method C		
	CP + EP	CP + EP + VC	CP + EP + EGCG	CP + EP	CP + EP + VC	CP + EP + EGCG	CP + EP	CP + EP + VC	CP + EP + EGCG
*Linear fitting*									
> 3 kDa	0.56	1.05	0.53	0.11	2.28	1.05	0.66	2.53	0.89
2–3 kDa	0.86	0.42	0.44	0.53	0.83	1.28	1.59	1.43	0.49
1–2 kDa	0.56	0.36	0.43	0.22	0.73	0.14	0.48	1.96	1.34
0.5–1 kDa	0.71	0.71	0.56	0.49	0.56	1.05	1.12	0.66	2.63
< 0.5 kDa	0.28	0.27	0.28	0.34	0.39	0.67	0.32	0.97	1.04
Mn	0.50	0.60	0.96	0.52	0.49	0.45	1.07	1.11	1.22
Mw	0.47	0.44	0.44	1.84	1.92	2.01	0.53	1.42	2.31
Mz	0.47	0.83	0.53	0.44	0.42	0.86	0.87	0.75	0.92
*Cubic fitting*									
> 3 kDa	1.13	1.11	0.56	1.05	0.5	0.11	0.63	0.48	0.84
2–3 kDa	0.52	0.49	0.86	0.42	0.44	0.53	0.40	0.36	0.56
1–2 kDa	0.50	0.60	0.56	0.36	0.4	0.22	1.04	1.89	1.82
0.5–1 kDa	0.67	1.42	0.81	1.37	0.25	1.59	2.50	2.49	1.28
< 0.5 kDa	1.84	1.92	0.73	0.22	0.40	0.36	0.56	1.43	1.12
Mn	0.11	0.24	0.38	1.86	0.43	0.43	0.13	0.21	0.33
Mw	0.73	0.49	0.52	1.07	0.89	1.40	1.22	1.12	1.13
Mz	0.44	0.69	0.18	0.36	0.50	0.40	0.41	1.00	0.78

### 3.2 Identification and Exclusion of Beverage Matrix Interference, and Verification of Peptide Profile Consistency

**FIGURE 2:**
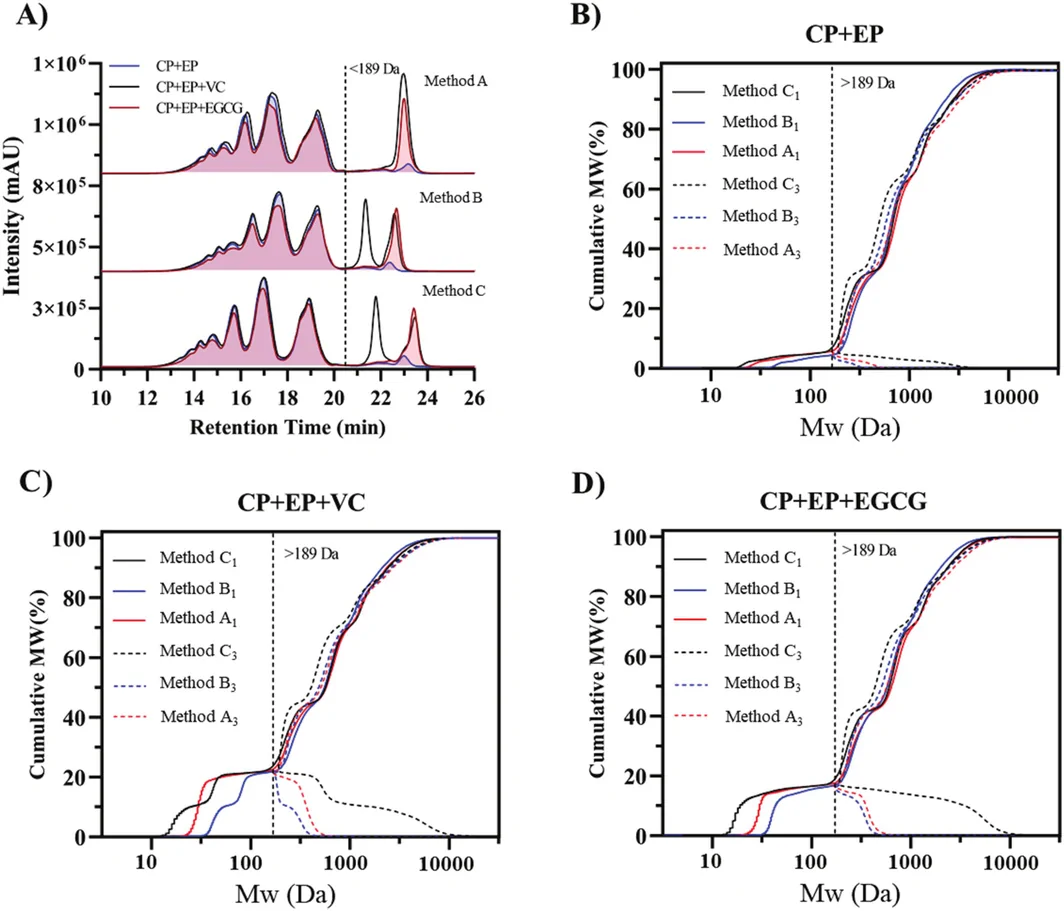
(A) Chromatograms of peptide powder and two peptide‐based beverages. (B–D) Cumulative molecular weight distribution of three samples under linear and cubic fitting for Methods A, B, and C, respectively. _1_linear fitting; _3_third‐order fitting.

To identify and exclude beverage matrix interference, the retention times of peptide standards and representative beverage excipients were compared under the three mobile phase conditions. As shown in Figure 1A, the smallest peptide standard GGG (189 Da) eluted at approximately 19.5 min across all three methods, with peak tailing extending to 20.0 min. Under the three detection methods, the molecular weight distribution profiles of pure peptide powder and the two beverages before 20.5 min were highly consistent (Figure 2A), indicating that peptide fractions predominantly eluted in this region. Theoretically, peaks eluting after 20.5 min correspond to compounds with molecular weights below 189 Da; however, one research suggests that this region may also contain aromatic excipients from the beverage matrix (Su et al. 2012). To verify this, representative beverage excipients were analyzed under identical chromatographic conditions. Vitamin C (VC) and epigallocatechin gallate (EGCG) both eluted after 20.5 min (Figure 1E,F), confirming that common functional additives in the beverage matrix appeared in the late‐eluting region.

**FIGURE 3:**
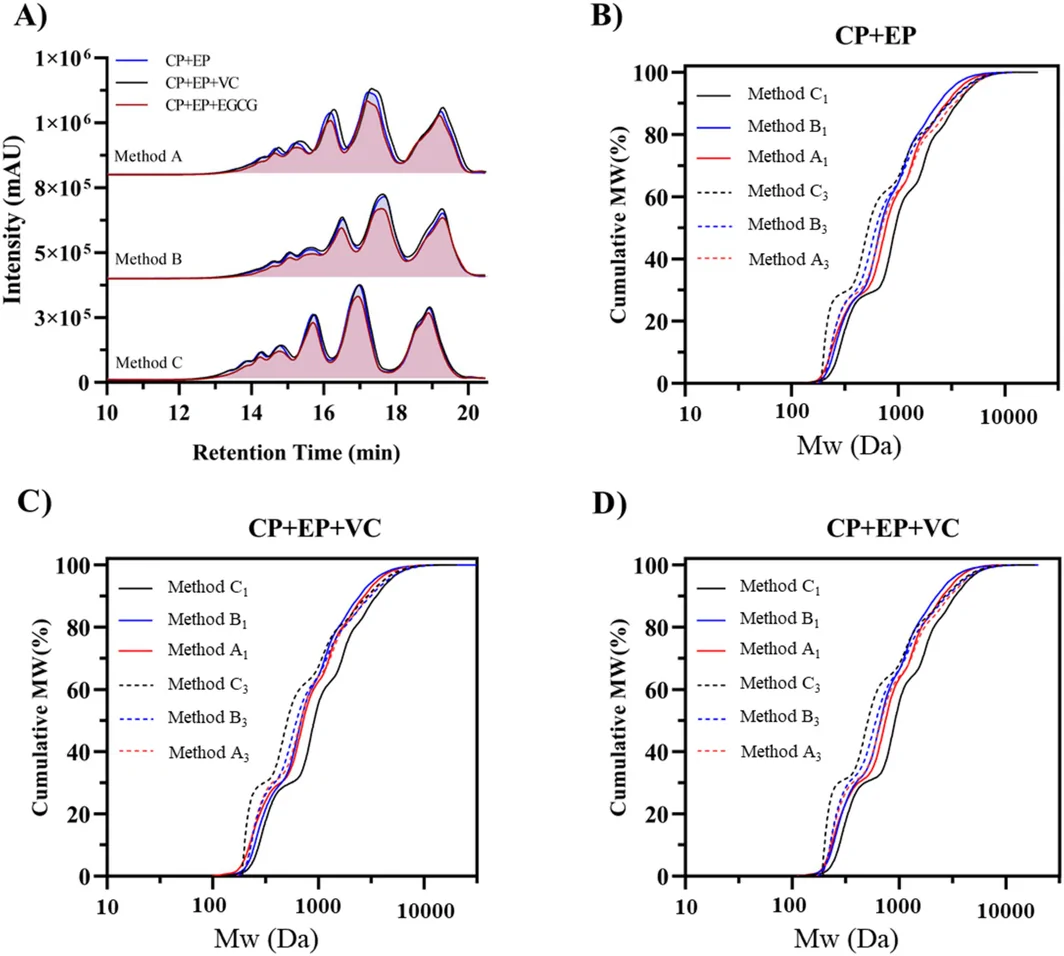
(A) Chromatograms of peptide powder and two peptide‐based beverages after interference peak removal for Methods A, B, and C. (B–D) Cumulative molecular weight distributions of the same samples after interference peak removal, analyzed by Methods A, B, and C under linear and cubic fitting. _1_linear fitting; _3_cubic fitting.

Cumulative molecular weight distributions of the three samples were further analyzed under different detection methods and fitting conditions. The results (Figure 2B–D) showed that the apparent proportion of the < 189 Da fraction was significantly higher in both beverages, reaching up to 20% of the total distribution, confirming that these signals mainly originated from non‐peptide substances and would affect the peptide molecular weight distribution results. Therefore, peaks eluting after 20.5 min were excluded from subsequent analyses. After excluding the interfering peaks, the chromatographic profiles of the three samples showed high overlap (Figure 3A). Statistical analysis of molecular weight distributions and parameters (Figure 3B–D) revealed that the relative deviations across all molecular weight ranges were below 10%, with no meaningful differences observed among the three samples under the same method. This indicated that the results accurately reflected the true molecular weight distribution of collagen peptides in the beverage matrix, consistent with that of pure peptide powder. This may be attributed to the fact that some excipients, such as polysaccharides, exhibited weak UV absorbance at 220 nm due to the absence of conjugated chromophores, and their contribution was therefore negligible, causing no significant interference with the molecular weight determination of collagen peptides.

**FIGURE 4:**
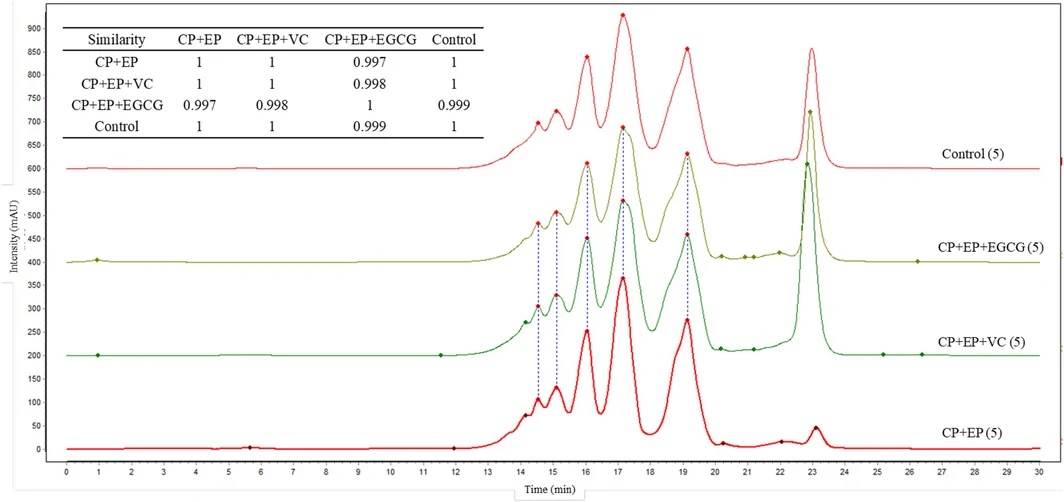
Similarity analysis of HPLC fingerprints for collagen powder and peptide‐based beverages using the Traditional Chinese Medicine Chromatographic Fingerprint Similarity Evaluation System (Version 2012.130723) under Method A.

To provide further validation, chromatographic fingerprint similarity analysis was performed (Cuadros‐Rodríguez et al. 2021). Using the pure peptide powder fingerprint as the reference, the similarity correlation coefficients between the two beverage fingerprints and the reference both exceeded 0.99 (Figure 4). This confirms from another dimension that the distribution of various molecular weight components is highly consistent between collagen peptide powder and the beverages.

### 3.3 Consistency of Molecular Weight Distribution Among Different Methods

**FIGURE 5:**
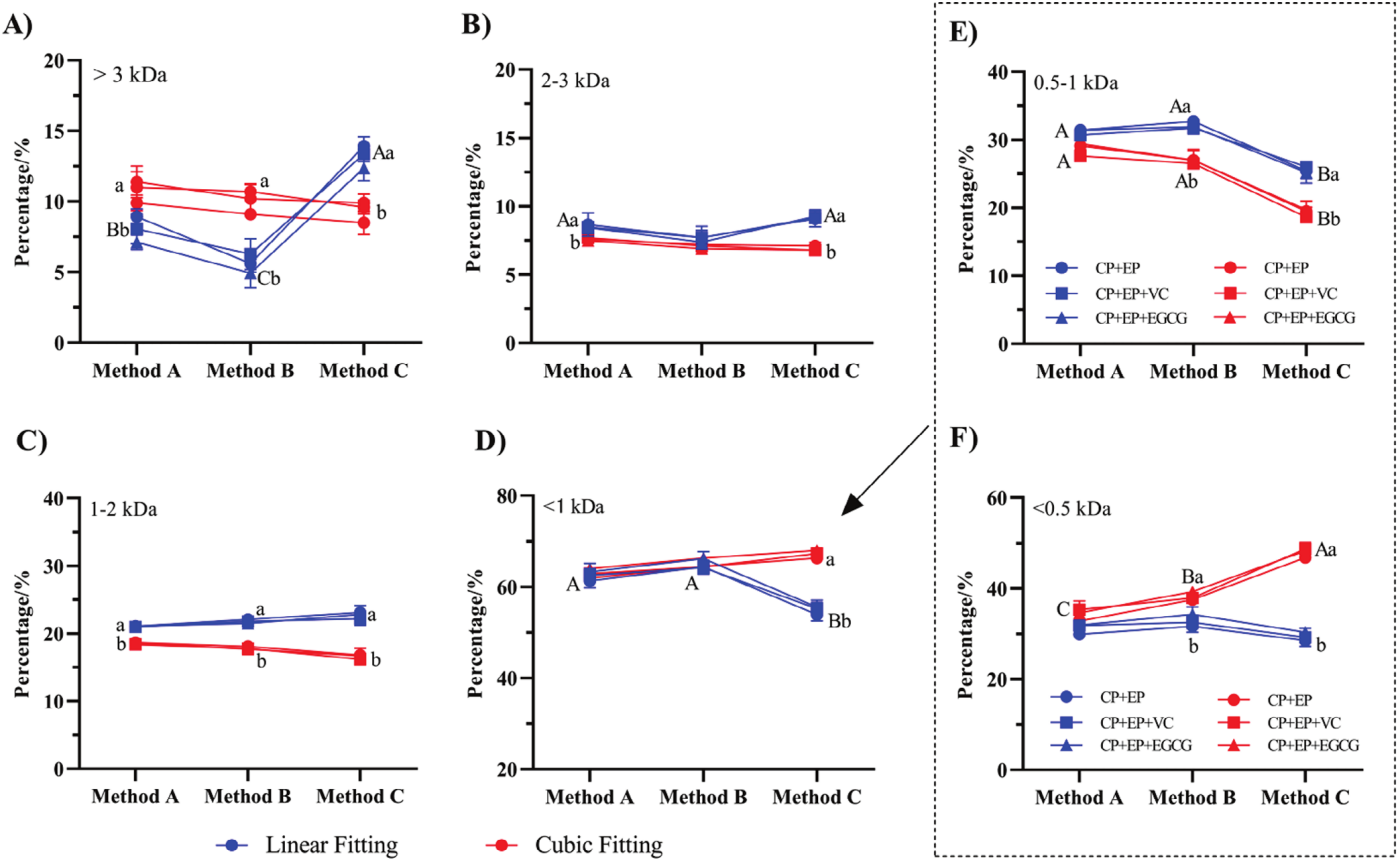
Molecular weight distribution percentages of peptide powder and two peptide‐based beverages analyzed by Methods A, B, and C, with linear and cubic fitting. (A) > 3 kDa, (B) 2–3 kDa, (C) 1–2 kDa, (D) < 1 kDa, (E) 0.5–1 kDa, and (F) < 0.5 kDa. Significant differences (RD > 10%) between methods for the same sample are indicated by uppercase letters (A/B/C), while differences between samples for the same method are indicated by lowercase letters (a/b/c). CP + EP: Peptide powder; CP + EP + VC: Peptide VC beverage; CP + EP + EGCG: Peptide EGCG beverage.

After excluding matrix interference, the molecular weight distribution results obtained by the three detection methods for peptide powder and beverage samples were systematically evaluated. As shown in Figure 5, under linear fitting, significant differences among the three methods were observed in the > 3 kDa range. In the 0.5–1 kDa range, Methods A and B showed no significant difference, but both differed significantly from Method C. Under cubic fitting, the differences among methods were markedly reduced, with significant differences remaining only in the 0.5–1 kDa and < 0.5 kDa ranges between Method C and Methods A/B.

Given that bioactive small peptides are primarily concentrated in the < 1 kDa range, data from sub‐ranges within this interval were combined for analysis (Lina et al. 2025; Villanueva et al. 2024). Under linear fitting, Methods A and B showed no significant difference, but both differed significantly from Method C. Under cubic fitting, no significant differences were observed among the three methods (RD < 10%). More importantly, the RSD values among methods under cubic fitting were all below 5%, indicating that cubic fitting effectively reduces systematic deviations among methods and improves consistency in this key molecular weight range.

Further comparison of the two fitting approaches within each method showed that Method A exhibited no significant differences in the < 1 kDa, 0.5–1 kDa, and < 0.5 kDa ranges. Method B showed no significant differences in the < 1 kDa and 2–3 kDa ranges. Method C showed significant differences across all ranges.

### 3.4 Consistency of Molecular Weight Parameters Among Different Methods

The physical and chemical properties of the polymers are directly correlated with the MW, molecular weight distribution, dispersity, and long‐chain branching (McCormick et al. 2003). Mn is associated with tensile strength, impact strength, and hardness, which is susceptible to low‐MW, and Mw and Mz are related to brittleness and rigidity, respectively, which are affected by high‐MW. The dispersion coefficient (*ρ*) and degree (*d*) of the sample have the same physical meaning to measure the broadness of the molecular weight distribution (Fijan et al. 2009; Stoleru et al. 2020). Generally speaking, the larger the polymer molecular weight dispersability, the greater the difference between the average MWs.

**FIGURE 6:**
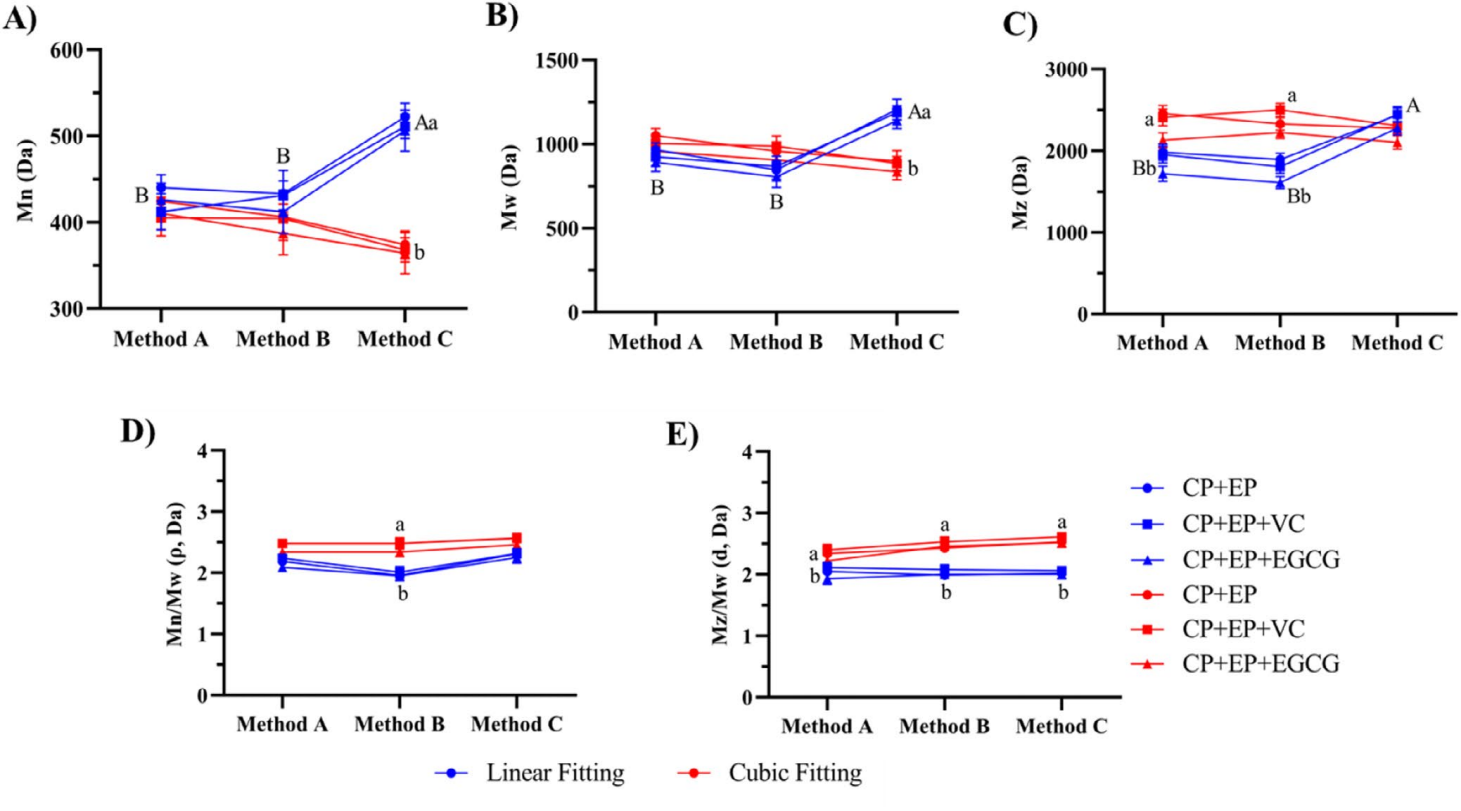
Molecular weight parameters (Mn, Mw, Mz, *ρ*, and *d*) of peptide powder and two peptide‐based beverages analyzed by Methods A, B, and C, with linear and cubic polynomial fitting. (A) Mn (number‐average molecular weight), (B) Mw (weight‐average molecular weight), (C) Mz (Z‐average molecular weight), (D) ρ (Mn/Mw), and (E) d (Mz/Mw). Significant differences (RD > 10%) between methods for the same sample are indicated by uppercase letters (A/B/C), while differences between samples for the same method are indicated by lowercase letters (a/b/c). CP + EP: Peptide powder; CP + EP + VC: Peptide VC beverage; CP + EP + EGCG: Peptide EGCG beverage.

Analysis of molecular weight parameters (Figure 6) showed that under linear fitting, significant differences were observed for Mn, Mw, and Mz between method C and methods A/B, while no significant differences were found for *ρ* and *d*. However, under cubic fitting, none of the five parameters (Mn, Mw, Mz, *ρ*, *d*) showed significant differences among the three methods. Additionally, the values of the key parameter Mw exhibited regular trends under different fitting approaches (linear: C > A > B; cubic: A > B > C). This shift clearly demonstrates that cubic fitting effectively eliminates statistical differences in most parameters across methods, significantly improving result consistency.

Further comparison of the two fitting approaches within each method showed that significant differences were observed in Mw and Mn for Method C, in Mz for Methods A and B, in *ρ* for Method B, and in *d* for all three methods.

### 3.5 Influence of Fitting Method on Measurement Results

**FIGURE 7:**
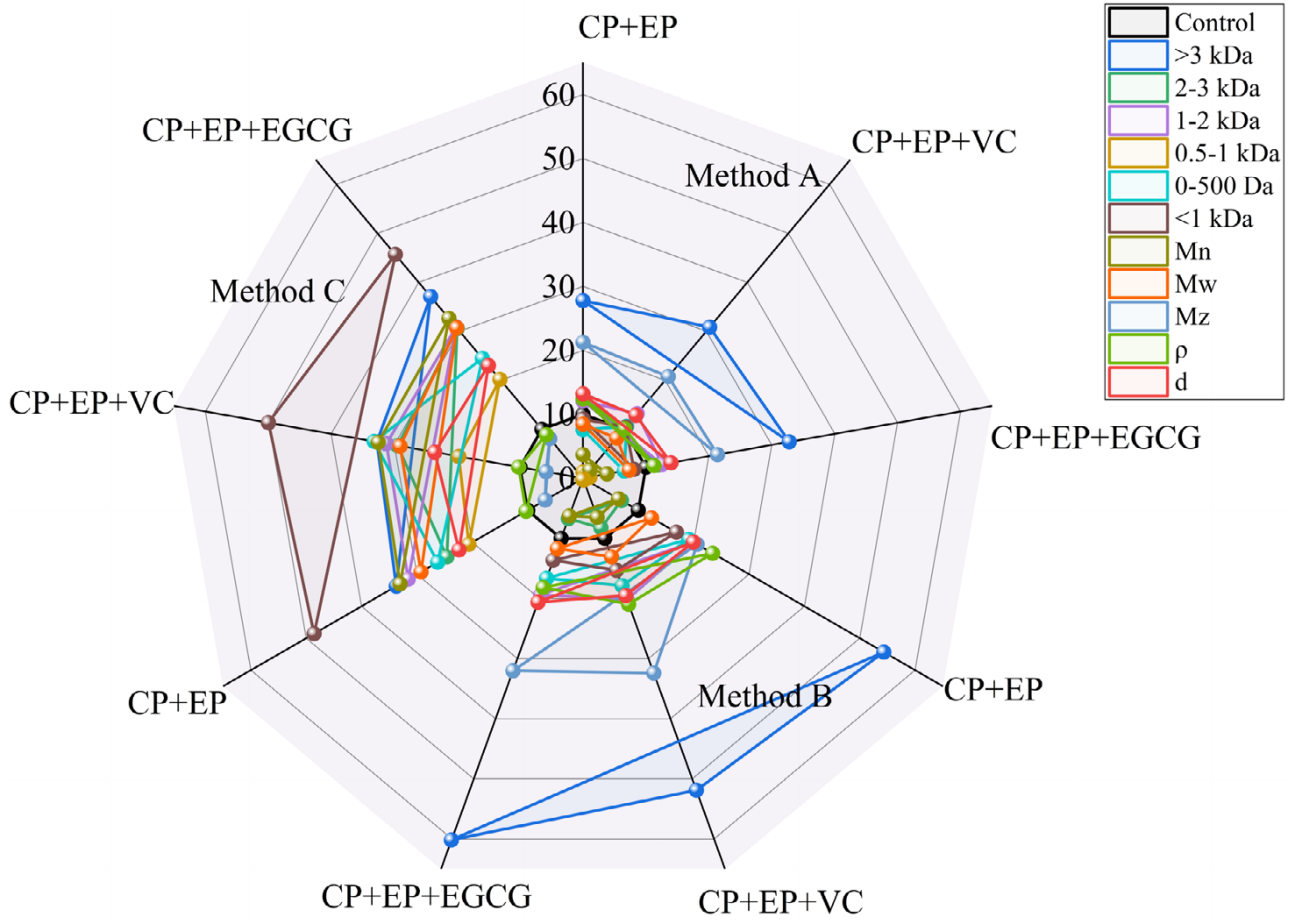
Relative deviation (RD, %) values for molecular weight distribution and parameters of three samples analyzed by Methods A, B, and C, comparing linear and cubic polynomial fitting. The RD value of 10% was set as the control for significance. CP + EP: Peptide powder; CP + EP + VC: Peptide VC beverage; CP + EP + EGCG: Peptide EGCG beverage.

To visually compare the effects of different fitting approaches on molecular weight distribution and parameter calculations, radar plot analysis was performed on the result differences between linear and cubic fitting for the three methods (Figure 7). Using a relative deviation (RD, %) threshold of 10% as the significance criterion, the overall deviation magnitude, excluding extreme deviations in individual parameters, followed the order: Method A (0%–15%) < Method B (5%–20%) < Method C (20%–35%). This indicates that Method A was least affected by the fitting approach, showing the best result consistency, while Method C was most significantly influenced.

## 4 Discussion

### 4.1 Development and Validation of a Matrix‐Resistant Analytical Strategy

A core challenge in analyzing functional ingredients in complex food systems is distinguishing the target analyte from the background matrix. This study successfully established a robust strategy to address this challenge for collagen peptides in beverages. The key innovation involves two steps: first, chromatographic identification and systematic exclusion of late‐eluting peaks (> 20.5 min) attributed to non‐peptide, UV‐absorbing excipients; second, application of cubic fitting for GPC data processing. The high consistency (RSD < 3%) of results across three distinct chromatographic methods (A, B, C) for both pure peptide powder and beverage matrices (Sections 3.1, 3.2, and 3.3) confirms the intrinsic precision of each method. More importantly, the subsequent alignment of molecular weight distribution profiles and key parameters (Mn, Mw, Mz) between the pure powder and the beverages after interference removal (Section 3.4) empirically validates the strategy's effectiveness. This demonstrates that the chosen chromatographic conditions (varying acetonitrile/water/TFA ratios) combined with the post‐acquisition data treatment effectively “filter out” the spectroscopic and hydrodynamic noise introduced by common beverage additives, isolating the signal specific to the collagen and elastin peptide population.

### 4.2 The Critical Role of Data Fitting on Inter‐Method Comparability

Achieving cross‐method and cross‐laboratory data comparability is fundamental for standardized quality control. After effectively eliminating matrix interference, we systematically evaluated the comparability of three mainstream detection methods and identified the data‐fitting model as the key variable determining result consistency.

All three methods demonstrated good reproducibility (RSD < 10%), providing a reliable foundation for horizontal comparison. However, analysis revealed that under linear fitting, significant differences persisted among the methods in key molecular weight ranges (e.g., > 3 kDa, 0.5–1 kDa, and < 0.5 kDa) and critical parameters (such as Mw and Mz). This is primarily because enzymatic hydrolysates exhibit broad polydispersity spanning multiple orders of magnitude in molecular weight, which produces a distinctly nonlinear relationship between retention time and lg(MW) that a cubic polynomial captures far more effectively than linear regression (Ó'Fágáin et al. 2017). Moreover, differences in chromatographic conditions (variations in mobile phase composition) further amplify the bias inherent in the linear model. In contrast, cubic fitting demonstrated excellent harmonizing capability. By more flexibly aligning with the molecular sieving characteristics of gel chromatography, this model effectively eliminated systematic deviations introduced by mobile phase differences, leading to highly consistent results across all three methods. This confirms that uniform adoption of cubic fitting is essential for achieving data comparability across different methods.

Based on these findings, we propose a practical analytical framework. If the industry adopts cubic fitting as a reference data processing approach, results from different detection methods will be directly comparable. Under linear fitting, Methods A and B remain consistent; under cubic fitting, all three methods yield consistent results. If fitting conditions cannot be unified in the short term, Method A, due to its better robustness across different fitting modes, can serve as the preferred reference method at this stage.

### 4.3 Compatibility Between Formula Optimization and Core Ingredient Analysis

**FIGURE 8:**
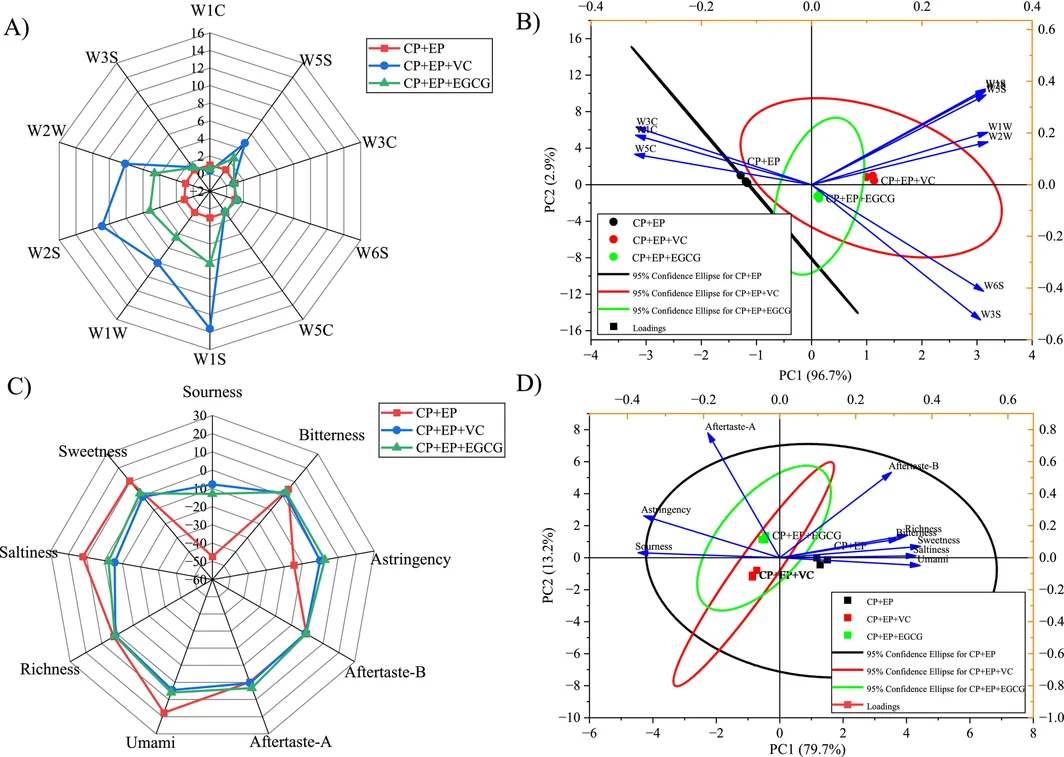
Radar charts and principal component analysis (PCA) of collagen powder and collagen‐based beverages. (A, B): E‐nose; (C, D): E‐tongue.

From a product‐development perspective, the robustness of the analytical method was further validated under the multidimensional influences of formula optimization. Physicochemical analysis showed that, compared with pure peptide powder, the addition of excipients such as VC, concentrated juice, chitooligosaccharide and pectin in the two beverages led to a decrease in pH and an increase in viscosity, which helps improve physical stability and extend shelf life (Table S2). Meanwhile, objective analysis of flavor profiles by electronic nose and tongue (Figure 8) revealed significant differences between the beverages and the peptide powder. The beverages showed stronger response signals on multiple sensors (e.g., W1S, W1W, W2S, W2W), reflecting a more complex volatile flavor composition (He et al. 2026; Zheng et al. 2025). Taste analysis also indicated that the distribution of umami, saltiness, sweetness and sourness in the beverages was more balanced and harmonious compared with the peptide powder. This demonstrates that product flavor can be systematically tailored through scientific formula adjustment to meet specific consumer preferences.

Critically, GPC results confirmed that the introduction of these water‐soluble excipients, along with the resulting changes in physicochemical properties and the successful shaping of the flavor system, did not interfere with the accurate determination of the molecular weight and distribution of collagen peptides. This finding clearly indicates that the stability of the functional ingredient is compatible with the adjustability of product sensory flavor in the formulation system. Enterprises can freely optimize flavor and adjust physical stability while ensuring consistent quality of the core peptide, thereby providing a solid practical basis for developing collagen peptide‐based beverage products that combine reliable efficacy, good stability, and excellent palatability.

### 4.4 Limitations and Future Perspectives

While this study establishes a robust analytical strategy, certain limitations should be acknowledged to guide future research. Firstly, all experiments were conducted on a single HPLC system, only verifying the instrument's precision, and no cross‐platform validation was carried out. Future research should be conducted on at least two different instrument platforms to confirm the universality of the research results. Secondly, the interference elimination strategy proposed in this study has not been tested in other beverage matrices. The main reason is that it is difficult to obtain peptide powder raw materials of the same batch as the commercial products for accurate verification. However, most common beverage components—including polysaccharides—have weak or negligible ultraviolet absorption at 220 nm due to the lack of conjugated chromophores or peptide bonds. Therefore, the applicability of this exclusion rule in other matrices needs to be verified on a case‐by‐case basis. For routine application to unknown matrices, spiking recovery experiments or blank matrix runs are recommended to confirm the absence of interference. Thirdly, the TSK‐GEL G2000 SWXL chromatographic column was used to investigate the overall distribution characteristics of fish‐derived collagen peptide molecular weights to meet the compliance requirements of low‐polymer peptide product quality control. However, this method has certain limitations in resolution and accuracy in the < 500 Da low‐molecular‐weight range. Within this range, the difference in peptide molecular weights is only equivalent to 1–2 amino acid residues, and the difference in fluid dynamic volume is extremely small, resulting in smooth chromatographic peaks. One research confirmed that the Superdex series chromatographic column has a better separation resolution for small molecule peptides than TSK‐GEL G2000 SWXL, but its single analysis time is up to 1.5 h, and the column equilibration time is long, making it difficult to meet the needs of high‐throughput daily quality control analysis (Feng et al. 2025). Therefore, for the fine characterization of the < 500 Da section, it is recommended to conduct cross‐validation by combining LC–MS/MS or dedicated small molecule SEC columns (such as Superdex 30 Increase). In addition, the nonlinear nature of GPC calibration curves is a general phenomenon in size‐exclusion chromatography rather than a condition‐specific observation. Held and Kilz (2009) systematically described the sigmoidal shape of GPC calibration curves and the necessity of polynomial fitting. Although the three‐fold fitting in this study shows good coordination ability, exploring other advanced data processing methods (such as multi‐point calibration, alternative nonlinear models) is expected to further optimize accuracy and expand the applicability of this analytical framework. Finally, future research can further explore the direct correlation between the precise peptide spectra obtained by this method and related in vitro biological activities (such as antioxidant capacity, collagen synthesis effect) or clinical efficacy endpoints, thereby achieving an effective connection between analytical consistency and functional predictability.

## 5 Conclusion

This study established a practical analytical framework for molecular weight analysis of fish‐derived collagen peptides in complex beverage matrices. Three key contributions are highlighted. First, a 20.5 min chromatographic cutoff was validated to exclude non‐peptide interference from beverage excipients, ensuring that the measured profiles accurately reflect the true peptide distribution. Second, cubic fitting was identified as superior to linear fitting in reducing inter‐method variability, achieving RSD below 5% in the range of < 1 kDa. Third, the framework was shown to be compatible with formulation optimization, allowing simultaneous control of peptide quality and sensory attributes. This study provides preliminary guidance for addressing the lack of detail in existing standards regarding data processing, and offers a practical strategy for cross‐comparison of analytical data to verify data accuracy. Nevertheless, further validation is still needed. Future work should extend this method to additional instrument platforms, different column batches, and a broader range of peptide‐based beverage matrices to validate its applicability and robustness, thereby providing a more comprehensive quality control tool for the industry.

## Author Contributions

**Mengmeng Feng:** conceptualization, investigation, validation, methodology, software, formal analysis, data curation, writing – original draft. **Yali Li:** methodology, investigation. **Yongkang Luo:** conceptualization, project administration. **Yanfei Jiang:** conceptualization, project administration. **Chunyue Zhao:** conceptualization, writing – review and editing.

## Funding

This work was supported by the National Key Research and Development Program of China for research and industrialization demonstration on key technologies of green processing and value‐added utilization of freshwater fish (2022YFD2100900).

## Consent

All the authors read and approved the final version of the manuscript.

## Conflicts of Interest

The authors declare no conflicts of interest.

## Supporting information


**Table S1:** Repeatability of retention times for calibration standards under three chromatographic methods.
**Table S2:** Physicochemical properties of collagen peptide powder and the two collagen peptide beverages.

## Data Availability

The data that support the findings of this study are available from the corresponding author upon reasonable request.
