# Supplementary material for: Developing an Analytical Framework for the Molecular Weight Analysis of Fish‐Derived Collagen Peptides in Beverages: A Multi‐Method Comparative Study
Source: Food Sci Nutr. 2026 Sep 18;14(9):e72386. doi: 10.1002/fsn3.72386 (PMC13589094; doi:10.1002/fsn3.72386)
Supplement: Supplementary file 1 [file FSN3-14-e72386-s001.docx]

Table S1 Repeatability of retention times for calibration standards under three chromatographic methods.

| Method | Calibration standards | RT_1_ (min) | RT_2_ (min) | RSD_RT_ (%) |
| --- | --- | --- | --- | --- |
| Method A | Cytochrome C (12,384 Da) | 11.92 | 11.930 | 0.059% |
|  | Aprotinin (6,511.44 Da) | 13.522 | 13.525 | 0.016% |
|  | Bacitracin (1,422.69 Da) | 16.093 | 16.087 | 0.026% |
|  | L-GSH (612.63 Da) | 16.978 | 16.981 | 0.012% |
|  | GPH (285.29 Da) | 18.783 | 18.777 | 0.023% |
|  | GGG (189 Da) | 19.544 | 19.548 | 0.014% |
| Method B | Cytochrome C (12,384 Da) | 11.884 | 11.879 | 0.030% |
|  | Aprotinin (6,511.44 Da) | 13.32 | 13.315 | 0.027% |
|  | Bacitracin (1,422.69 Da) | 15.547 | 15.542 | 0.023% |
|  | GGTA (451.48 Da) | 17.275 | 17.281 | 0.025% |
|  | GGG (189 Da) | 19.539 | 19.533 | 0.022% |
| Method C | Cytochrome C (12,384 Da) | 11.789 | 11.795 | 0.036% |
|  | Aprotinin (6,511.44 Da) | 13.321 | 13.327 | 0.032% |
|  | Bacitracin (1,422.69 Da) | 15.17 | 15.165 | 0.023% |
|  | GGTA (451.48 Da) | 17.02 | 17.014 | 0.025% |
|  | GGG (189 Da) | 19.463 | 19.457 | 0.022% |

Note: RT₁ and RT₂ represent the retention times of the first and last injections of each calibration standard within a given analytical sequence, respectively. RSD_RT_ (%) was calculated from duplicate measurements (n = 2). Method A: 30/70/0.1 (v/v/v) per Q/HNHY 002-2021. Method B: acetonitrile/water/TFA = 40/60/0.1 (v/v/v) per GB 31645-2018; Method C: 45/55/0.1 (v/v/v) per GB/T 22729-2008; L-GSH: L‑oxidized glutathione; GPH: Gly‑Pro‑Hyp; GGTA: Gly‑Gly‑Tyr‑Arg; GGG: Gly‑Gly‑Gly.

Table S2 Physicochemical properties of collagen peptide powder and the two collagen peptide beverages.

| Variable | Collagen  powder | Collagen VC beverage | Collagen EGCG beverage |
| --- | --- | --- | --- |
| Density (g/mL) | 1.0394 ± 0.00^c^ | 1.0658 ± 0.00^b^ | 1.0805 ± 0.00^a^ |
| Viscosity (cP) | 6.95 ± 0.03^b^ | 29.3 ± 0.02^a^ | 29.5 ± 0.02^a^ |
| pH | 6.36 ± 0.01^a^ | 4.06 ± 0.01^b^ | 4.01 ± 0.01^c^ |
| Soluble Solids (Brix, %) | 11.1 ± 0.02^c^ | 21.71 ± 0.03^b^ | 22.15 ± 0.04^a^ |
| Titratable Acidity (% citric acid, g/kg) | / | 14.71 ± 0.04^b^ | 15.63 ± 0.06^a^ |

Note:

Data are expressed as mean ± standard deviation (SD) from three independent measurements (n = 3). Density was measured using an electronic analytical balance (FA2104, Sunny Hengping, China); viscosity was determined using a rotational viscometer (ROTAVISC LO-VI S000, IKA, Germany); pH was measured with a pH meter (ST20, OHAUS, USA); soluble solids content was determined using a portable refractometer (0–50%, LICHEN, China); and titratable acidity was determined by titration with 0.1 M NaOH using an automatic potentiometric titrator (ZD-2, LEICI, China). Within each row, values with different superscript letters (a, b, c) are significantly different (p < 0.05) according to one‑way ANOVA followed by Tukey's post‑hoc test. "/" indicates not determined (collagen peptide powder contains no organic acids). VC: vitamin C; EGCG: epigallocatechin gallate.
